# Quantitative and Qualitative Approaches to Generalization and Replication–A Representationalist View

**DOI:** 10.3389/fpsyg.2021.605191

**Published:** 2021-02-05

**Authors:** Matthias Borgstede, Marcel Scholz

**Affiliations:** Foundations of Education, University of Bamberg, Bamberg, Germany

**Keywords:** qualitative research, representational measurement, research methodology, modal logic, generalizability, replication crisis

## Abstract

In this paper, we provide a re-interpretation of qualitative and quantitative modeling from a representationalist perspective. In this view, both approaches attempt to construct abstract representations of empirical relational structures. Whereas quantitative research uses variable-based models that abstract from individual cases, qualitative research favors case-based models that abstract from individual characteristics. Variable-based models are usually stated in the form of quantified sentences (scientific laws). This syntactic structure implies that sentences about individual cases are derived using deductive reasoning. In contrast, case-based models are usually stated using context-dependent existential sentences (qualitative statements). This syntactic structure implies that sentences about other cases are justifiable by inductive reasoning. We apply this representationalist perspective to the problems of generalization and replication. Using the analytical framework of modal logic, we argue that the modes of reasoning are often not only applied to the context that has been studied empirically, but also on a between-contexts level. Consequently, quantitative researchers mostly adhere to a top-down strategy of generalization, whereas qualitative researchers usually follow a bottom-up strategy of generalization. Depending on which strategy is employed, the role of replication attempts is very different. In deductive reasoning, replication attempts serve as empirical tests of the underlying theory. Therefore, failed replications imply a faulty theory. From an inductive perspective, however, replication attempts serve to explore the scope of the theory. Consequently, failed replications do not question the theory *per se*, but help to shape its boundary conditions. We conclude that quantitative research may benefit from a bottom-up generalization strategy as it is employed in most qualitative research programs. Inductive reasoning forces us to think about the boundary conditions of our theories and provides a framework for generalization beyond statistical testing. In this perspective, failed replications are just as informative as successful replications, because they help to explore the scope of our theories.

## Introduction

Qualitative and quantitative research strategies have long been treated as opposing paradigms. In recent years, there have been attempts to integrate both strategies. These “mixed methods” approaches treat qualitative and quantitative methodologies as complementary, rather than opposing, strategies (Creswell, 2015). However, whilst acknowledging that both strategies have their benefits, this “integration” remains purely pragmatic. Hence, mixed methods methodology does not provide a conceptual unification of the two approaches.

Lacking a common methodological background, qualitative and quantitative research methodologies have developed rather distinct standards with regard to the aims and scope of empirical science (Freeman et al., 2007). These different standards affect the way researchers handle contradictory empirical findings. For example, many empirical findings in psychology have failed to replicate in recent years (Klein et al., 2014; Open Science, Collaboration, 2015). This “replication crisis” has been discussed on statistical, theoretical and social grounds and continues to have a wide impact on quantitative research practices like, for example, open science initiatives, pre-registered studies and a re-evaluation of statistical significance testing (Everett and Earp, 2015; Maxwell et al., 2015; Shrout and Rodgers, 2018; Trafimow, 2018; Wiggins and Chrisopherson, 2019).

However, qualitative research seems to be hardly affected by this discussion. In this paper, we argue that the latter is a direct consequence of how the concept of generalizability is conceived in the two approaches. Whereas most of quantitative psychology is committed to a top-down strategy of generalization based on the idea of random sampling from an abstract population, qualitative studies usually rely on a bottom-up strategy of generalization that is grounded in the successive exploration of the field by means of theoretically sampled cases.

Here, we show that a common methodological framework for qualitative and quantitative research methodologies is possible. We accomplish this by introducing a formal description of quantitative and qualitative models from a representationalist perspective: both approaches can be reconstructed as special kinds of representations for empirical relational structures. We then use this framework to analyze the generalization strategies used in the two approaches. These turn out to be logically independent of the type of model. This has wide implications for psychological research. First, a top-down generalization strategy is compatible with a qualitative modeling approach. This implies that mainstream psychology may benefit from qualitative methods when a numerical representation turns out to be difficult or impossible, without the need to commit to a “qualitative” philosophy of science. Second, quantitative research may exploit the bottom-up generalization strategy that is inherent to many qualitative approaches. This offers a new perspective on unsuccessful replications by treating them not as scientific failures, but as a valuable source of information about the scope of a theory.

## The Quantitative Strategy–Numbers and Functions

**Figure 1:**
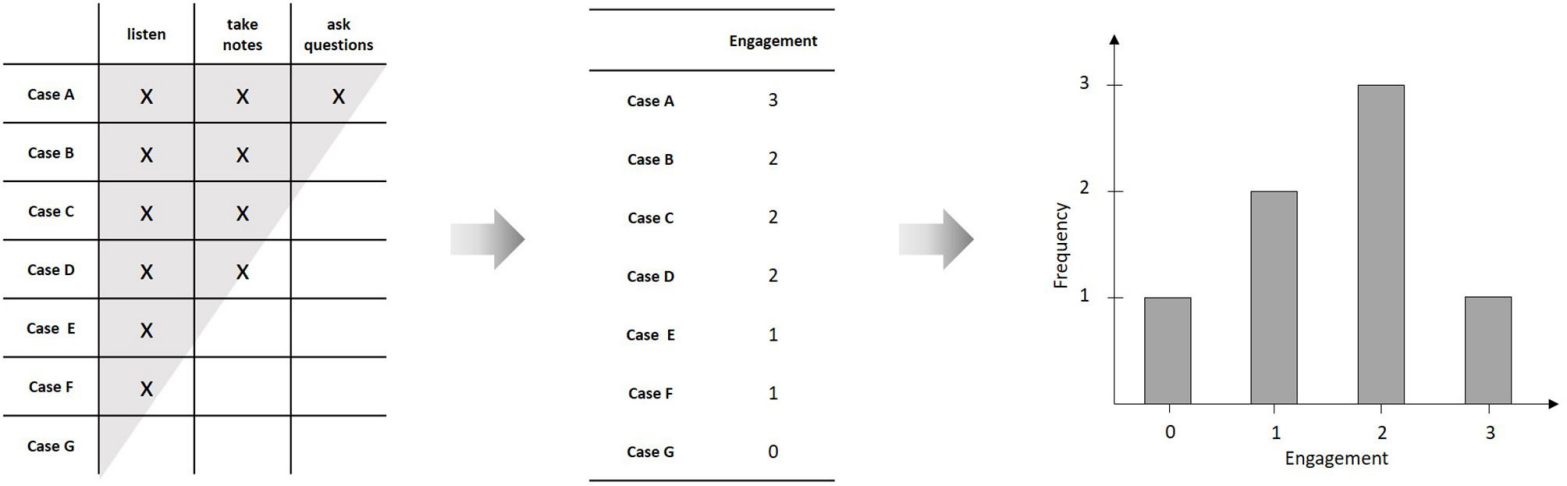
Constructing a numerical representation from an empirical relational structure; Due to the unique ordering of persons with regard to behaviors (indicated by the triangular shape of the relation), it is possible to construct a Guttman scale by assigning a number to each of the individuals, representing the number of relevant behaviors shown by the individual. The resulting variable (“engagement”) can then be described by means of statistical analyses, like, e.g., plotting the frequency distribution.

Quantitative science is about finding valid mathematical representations for empirical phenomena. In most cases, these mathematical representations have the form of functional relations between a set of variables. One major challenge of quantitative modeling consists in constructing valid measures for these variables. Formally, to measure a variable means to construct a numerical representation of the underlying empirical relational structure (Krantz et al., 1971). For example, take the behaviors of a group of students in a classroom: “to listen,” “to take notes,” and “to ask critical questions.” One may now ask whether is possible to assign numbers to the students, such that the relations between the assigned numbers are of the same kind as the relations between the values of an underlying variable, like e.g., “engagement.” The observed behaviors in the classroom constitute an empirical relational structure, in the sense that for every student-behavior tuple, one can observe whether it is true or not. These observations can be represented in a person × behavior matrix^1^ (compare Figure 1). Given this relational structure satisfies certain conditions (i.e., the *axioms* of a measurement model), one can assign numbers to the students and the behaviors, such that the relations between the numbers resemble the corresponding numerical relations. For example, if there is a unique ordering in the empirical observations with regard to which person shows which behavior, the assigned numbers have to constitute a corresponding unique ordering, as well. Such an ordering coincides with the person × behavior matrix forming a triangle shaped relation and is formally represented by a Guttman scale (Guttman, 1944). There are various measurement models available for different empirical structures (Suppes et al., 1971). In the case of probabilistic relations, Item-Response models may be considered as a special kind of measurement model (Borsboom, 2005).

**Figure 2:**
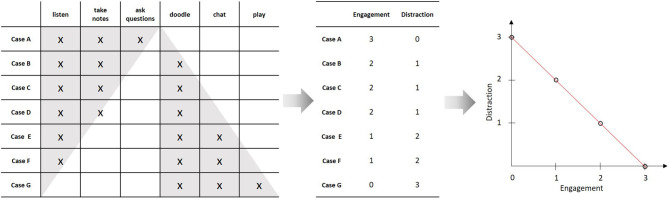
Constructing a numerical model from an empirical relational structure; Since there are two distinct classes of behaviors that each form a Guttman scale, it is possible to assign two numbers to each individual, correspondingly. The resulting variables (“engagement” and “distraction”) can then be related by a mathematical function, which is indicated by the scatterplot and red line on the right hand side.

Although essential, measurement is only the first step of quantitative modeling. Consider a slightly richer empirical structure, where we observe three additional behaviors: “to doodle,” “to chat,” and “to play.” Like above, one may ask, whether there is a unique ordering of the students with regard to these behaviors that can be represented by an underlying variable (i.e., whether the matrix forms a Guttman scale). If this is the case, we may assign corresponding numbers to the students and call this variable “distraction.” In our example, such a representation is possible. We can thus assign two numbers to each student, one representing his or her “engagement” and one representing his or her “distraction” (compare Figure 2). These measurements can now be used to construct a quantitative model by relating the two variables by a mathematical function. In the simplest case, this may be a linear function. This functional relation constitutes a quantitative model of the empirical relational structure under study (like, e.g., linear regression). Given the model equation and the rules for assigning the numbers (i.e., the instrumentations of the two variables), the set of admissible empirical structures is limited from all possible structures to a rather small subset. This constitutes the empirical content of the model^2^ (Popper, 1935).

## The Qualitative Strategy–Categories and Typologies

The predominant type of analysis in qualitative research consists in category formation. By constructing descriptive systems for empirical phenomena, it is possible to analyze the underlying empirical structure at a higher level of abstraction. The resulting categories (or types) constitute a conceptual frame for the interpretation of the observations. Qualitative researchers differ considerably in the way they collect and analyze data (Miles et al., 2014). However, despite the diverse research strategies followed by different qualitative methodologies, from a formal perspective, most approaches build on some kind of categorization of cases that share some common features. The process of category formation is essential in many qualitative methodologies, like, for example, qualitative content analysis, thematic analysis, grounded theory (see Flick, 2014 for an overview). Sometimes these features are directly observable (like in our classroom example), sometimes they are themselves the result of an interpretative process (e.g., Scheunpflug et al., 2016).

In contrast to quantitative methodologies, there have been little attempts to formalize qualitative research strategies (compare, however, Rihoux and Ragin, 2009). However, there are several statistical approaches to non-numerical data that deal with constructing abstract categories and establishing relations between these categories (Agresti, 2013). Some of these methods are very similar to qualitative category formation on a conceptual level. For example, cluster analysis groups cases into homogenous categories (clusters) based on their similarity on a distance metric.

Although category formation can be formalized in a mathematically rigorous way (Ganter and Wille, 1999), qualitative research hardly acknowledges these approaches.^3^ However, in order to find a common ground with quantitative science, it is certainly helpful to provide a formal interpretation of category systems.

**Figure 3:**
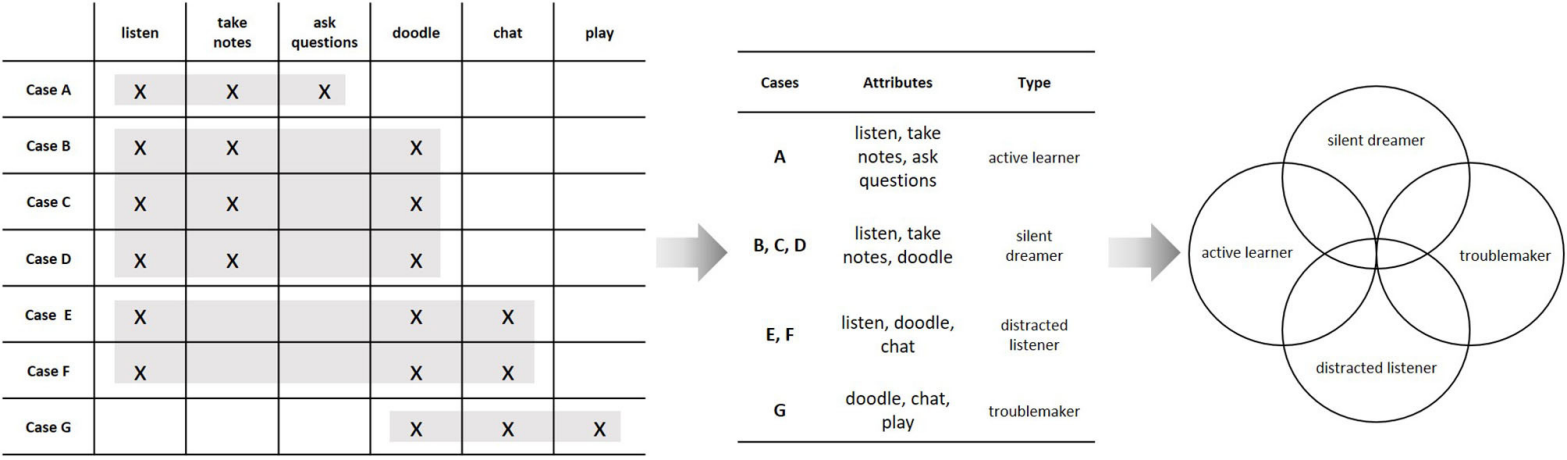
Constructing a conceptual model from an empirical relational structure; Individual behaviors are grouped to form abstract *types* based on them being shared among a specific subset of the cases. Each type constitutes a set of specific commonalities of a class of individuals (this is indicated by the rectangles on the left hand side). The resulting types (“active learner,” “silent dreamer,” “distracted listener,” and “troublemaker”) can then be related to one another to explicate their semantic and empirical overlap, as indicated by the Venn-diagram on the right hand side.

Let us reconsider the above example of students in a classroom. The quantitative strategy was to assign numbers to the students with regard to variables and to relate these variables via a mathematical function. We can analyze the same empirical structure by grouping the behaviors to form abstract categories. If the aim is to construct an empirically valid category system, this grouping is subject to constraints, analogous to those used to specify a measurement model. The first and most important constraint is that the behaviors must form equivalence classes, i.e., within categories, behaviors need to be equivalent, and across categories, they need to be distinct (formally, the relational structure must obey the axioms of an equivalence relation). When objects are grouped into equivalence classes, it is essential to specify the criterion for empirical equivalence. In qualitative methodology, this is sometimes referred to as the *tertium comparationis* (Flick, 2014). One possible criterion is to group behaviors such that they constitute a set of specific common attributes of a group of people. In our example, we might group the behaviors “to listen,” “to take notes,” and “to doodle,” because these behaviors are common to the cases B, C, and D, and they are also specific for these cases, because no other person shows this particular combination of behaviors. The set of common behaviors then forms an abstract concept (e.g., “moderate distraction”), while the set of persons that show this configuration form a type (e.g., “the silent dreamer”). Formally, this means to identify the *maximal rectangles* in the underlying empirical relational structure (see Figure 3). This procedure is very similar to the way we constructed a Guttman scale, the only difference being that we now use different aspects of the empirical relational structure.^4^ In fact, the set of maximal rectangles can be determined by an automated algorithm (Ganter, 2010), just like the dimensionality of an empirical structure can be explored by psychometric scaling methods. Consequently, we can identify the empirical content of a category system or a typology as the set of empirical structures that conforms to it.^5^ Whereas the quantitative strategy was to search for scalable sub-matrices and then relate the constructed variables by a mathematical function, the qualitative strategy is to construct an empirical typology by grouping cases based on their specific similarities. These types can then be related to one another by a conceptual model that describes their semantic and empirical overlap (see Figure 3, right hand side).

## Variable-Based Models and Case-Based Models

In the previous section, we have argued that qualitative category formation and quantitative measurement can both be characterized as methods to construct abstract representations of empirical relational structures. Instead of focusing on different philosophical approaches to empirical science, we tried to stress the formal similarities between both approaches. However, it is worth also exploring the dissimilarities from a formal perspective.

Following the above analysis, the quantitative approach can be characterized by the use of variable-based models, whereas the qualitative approach is characterized by case-based models (Ragin, 1987). Formally, we can identify the rows of an empirical person × behavior matrix with a person-space, and the columns with a corresponding behavior-space. A variable-based model abstracts from the single individuals in a person-space to describe the structure of behaviors on a population level. A case-based model, on the contrary, abstracts from the single behaviors in a behavior-space to describe individual case configurations on the level of abstract categories (see Table 1).

**Table 1:** Variable-based models and case-based models.

**Variable-based models**	**Case-based models**
Primarily used in quantitative research	Primarily used in qualitative research
Description of behaviors based on person-space	Description of individuals based on behavior-space
Abstraction from individuals to populations	Abstraction from behaviors to categories
Syntactic form: ∀_*i*_:*y*_*i*_ = *f*(*x*_*i*_**)**	Syntactic form: ∃_*i*_ : *XYZ*_*i*_
Application to behaviors: induction	Application to behaviors: deduction
Application to cases: deduction	Application to cases: induction

From a representational perspective, there is no a priori reason to favor one type of model over the other. Both approaches provide different analytical tools to construct an abstract representation of an empirical relational structure. However, since the two modeling approaches make use of different information (person-space vs. behavior-space), this comes with some important implications for the researcher employing one of the two strategies. These are concerned with the role of deductive and inductive reasoning.

In variable-based models, empirical structures are represented by functional relations between variables. These are usually stated as scientific laws (Carnap, 1928). Formally, these laws correspond to logical expressions of the form

$$\begin{array}{l} \forall_{i}\;:\;y_{i}=f\left(x_{i}\right) \end{array}$$

In plain text, this means that *y* is a function of *x* for all objects *i* in the relational structure under consideration. For example, in the above example, one may formulate the following law: for all students in the classroom it holds that “distraction” is a monotone decreasing function of “engagement.” Such a law can be used to derive predictions for single individuals by means of logical deduction: if the above law applies to all students in the classroom, it is possible to calculate the expected distraction from a student's engagement. An empirical observation can now be evaluated against this prediction. If the prediction turns out to be false, the law can be refuted based on the principle of falsification (Popper, 1935). If a scientific law repeatedly withstands such empirical tests, it may be considered to be valid with regard to the relational structure under consideration.

In case-based models, there are no laws about a population, because the model does not abstract from the cases but from the observed behaviors. A case-based model describes the underlying structure in terms of existential sentences. Formally, this corresponds to a logical expression of the form

$$\begin{array}{l} \exists_{i}\;:\;XYZ_{i} \end{array}$$

In plain text, this means that there is at least one case *i* for which the condition *XYZ* holds. For example, the above category system implies that there is at least one active learner. This is a statement about a singular observation. It is impossible to deduce a statement about another person from an existential sentence like this. Therefore, the strategy of falsification cannot be applied to test the model's validity in a specific context. If one wishes to generalize to other cases, this is accomplished by inductive reasoning, instead. If we observed one person that fulfills the criteria of calling him or her an active learner, we can hypothesize that there may be other persons that are identical to the observed case in this respect. However, we do not arrive at this conclusion by logical deduction, but by induction.

Despite this important distinction, it would be wrong to conclude that variable-based models are intrinsically deductive and case-based models are intrinsically inductive.^6^ Both types of reasoning apply to both types of models, but on different levels. Based on a person-space, in a variable-based model one can use deduction to derive statements about individual persons from abstract population laws. There is an analogous way of reasoning for case-based models: because they are based on a behavior space, it is possible to deduce statements about singular behaviors. For example, if we know that Peter is an active learner, we can deduce that he takes notes in the classroom. This kind of deductive reasoning can also be applied on a higher level of abstraction to deduce thematic categories from theoretical assumptions (Braun and Clarke, 2006). Similarly, there is an analog for inductive generalization from the perspective of variable-based modeling: since the laws are only quantified over the person-space, generalizations to other behaviors rely on inductive reasoning. For example, it is plausible to assume that highly engaged students tend to do their homework properly–however, in our example this behavior has never been observed. Hence, in variable-based models we usually generalize to other behaviors by means of induction. This kind of inductive reasoning is very common when empirical results are generalized from the laboratory to other behavioral domains.

Although inductive and deductive reasoning are used in qualitative and quantitative research, it is important to stress the different roles of induction and deduction when models are applied to cases. A variable-based approach implies to draw conclusions about cases by means of logical deduction; a case-based approach implies to draw conclusions about cases by means of inductive reasoning. In the following, we build on this distinction to differentiate between qualitative (bottom-up) and quantitative (top-down) strategies of generalization.

## Generalization and the Problem of Replication

We will now extend the formal analysis of quantitative and qualitative approaches to the question of generalization and replicability of empirical findings. For this sake, we have to introduce some concepts of formal logic. Formal logic is concerned with the validity of arguments. It provides conditions to evaluate whether certain sentences (conclusions) can be derived from other sentences (premises). In this context, a *theory* is nothing but a set of sentences (also called axioms). Formal logic provides tools to derive new sentences that must be true, given the axioms are true (Smith, 2020). These derived sentences are called *theorems* or, in the context of empirical science, *predictions* or *hypotheses*. On the syntactic level, the rules of logic only state how to evaluate the truth of a sentence relative to its premises. Whether or not sentences are actually true, is formally specified by logical semantics.

On the semantic level, formal logic is intrinsically linked to set-theory. For example, a logical statement like “all dogs are mammals,” is true if and only if the set of dogs is a subset of the set of mammals. Similarly, the sentence “all chatting students doodle” is true if and only if the set of chatting students is a subset of the set of doodling students (compare Figure 3). Whereas, the first sentence is analytically true due to the way we define the words “dog” and “mammal,” the latter can be either true or false, depending on the relational structure we actually observe. We can thus interpret an empirical relational structure as the truth criterion of a scientific theory. From a logical point of view, this corresponds to the semantics of a theory. As shown above, variable-based and case-based models both give a formal representation of the same kinds of empirical structures. Accordingly, both types of models can be stated as formal theories. In the variable-based approach, this corresponds to a set of scientific laws that are quantified over the members of an abstract population (these are the axioms of the theory). In the case-based approach, this corresponds to a set of abstract existential statements about a specific class of individuals.

In contrast to mathematical axiom systems, empirical theories are usually not considered to be *necessarily* true. This means that even if we find no evidence against a theory, it is still possible that it is actually wrong. We may know that a theory is valid in some contexts, yet it may fail when applied to a new set of behaviors (e.g., if we use a different instrumentation to measure a variable) or a new population (e.g., if we draw a new sample).

From a logical perspective, the possibility that a theory may turn out to be false stems from the problem of *contingency*. A statement is contingent, if it is both, possibly true and possibly false. Formally, we introduce two *modal* operators: □ to designate logical necessity, and ◇ to designate logical possibility. Semantically, these operators are very similar to the existential quantifier, ∃, and the universal quantifier, ∀. Whereas ∃ and ∀ refer to the individual objects within one relational structure, the modal operators □ and ◇ range over so-called *possible worlds*: a statement is possibly true, if and only if it is true in at least one accessible possible world, and a statement is necessarily true if and only if it is true in every accessible possible world (Hughes and Cresswell, 1996). Logically, possible worlds are mathematical abstractions, each consisting of a relational structure. Taken together, the relational structures of all accessible possible worlds constitute the formal semantics of necessity, possibility and contingency.^7^

In the context of an empirical theory, each possible world may be identified with an empirical relational structure like the above classroom example. Given the set of intended applications of a theory (the *scope* of the theory, one may say), we can now construct possible world semantics for an empirical theory: each intended application of the theory corresponds to a possible world. For example, a quantified sentence like “all chatting students doodle” may be true in one classroom and false in another one. In terms of possible worlds, this would correspond to a statement of contingency: “it is possible that all chatting students doodle in one classroom, and it is possible that they don't in another classroom.” Note that in the above expression, “all students” refers to the students in only one possible world, whereas “it is possible” refers to the fact that there is at least one possible world for each of the specified cases.

To apply these possible world semantics to quantitative research, let us reconsider how generalization to other cases works in variable-based models. Due to the syntactic structure of quantitative laws, we can deduce predictions for singular observations from an expression of the form ∀_*i*_ : *y*_*i*_ = *f*(*x*_*i*_). Formally, the logical quantifier ∀ ranges only over the objects of the corresponding empirical relational structure (in our example this would refer to the students in the observed classroom). But what if we want to generalize beyond the empirical structure we actually observed? The standard procedure is to assume an infinitely large, abstract population from which a random sample is drawn. Given the truth of the theory, we can deduce predictions about what we may observe in the sample. Since usually we deal with probabilistic models, we can evaluate our theory by means of the conditional probability of the observations, given the theory holds. This concept of conditional probability is the foundation of statistical significance tests (Hogg et al., 2013), as well as Bayesian estimation (Watanabe, 2018). In terms of possible world semantics, the random sampling model implies that all possible worlds (i.e., all intended applications) can be conceived as empirical sub-structures from a greater population structure. For example, the empirical relational structure constituted by the observed behaviors in a classroom would be conceived as a sub-matrix of the population person × behavior matrix. It follows that, if a scientific law is true in the population, it will be true in all possible worlds, i.e., it will be *necessarily* true. Formally, this corresponds to an expression of the form

$$\begin{array}{l} □\left(\forall_{i}\;:\;y_{i}=f\left(x_{i}\right)\right) \end{array}$$

The statistical generalization model thus constitutes a top-down strategy for dealing with individual contexts that is analogous to the way variable-based models are applied to individual cases (compare Table 1). Consequently, if we apply a variable-based model to a new context and find out that it does not fit the data (i.e., there is a statistically significant deviation from the model predictions), we have reason to doubt the validity of the theory. This is what makes the problem of low replicability so important: we observe that the predictions are wrong in a new study; and because we apply a top-down strategy of generalization to contexts beyond the ones we observed, we see our whole theory at stake.

Qualitative research, on the contrary, follows a different strategy of generalization. Since case-based models are formulated by a set of context-specific existential sentences, there is no need for universal truth or necessity. In contrast to statistical generalization to other cases by means of random sampling from an abstract population, the usual strategy in case-based modeling is to employ a bottom-up strategy of generalization that is analogous to the way case-based models are applied to individual cases. Formally, this may be expressed by stating that the observed qualia exist in at least one possible world, i.e., the theory is *possibly* true:

$$\begin{array}{l} ◇\left(\exists_{i}\;:\;XYZ_{i}\right) \end{array}$$

This statement is analogous to the way we apply case-based models to individual cases (compare Table 1). Consequently, the set of intended applications of the theory does not follow from a sampling model, but from theoretical assumptions about which cases may be similar to the observed cases with respect to certain relevant characteristics. For example, if we observe that certain behaviors occur together in one classroom, following a bottom-up strategy of generalization, we will hypothesize why this might be the case. If we do not replicate this finding in another context, this does not question the model itself, since it was a context-specific theory all along. Instead, we will revise our hypothetical assumptions about why the new context is apparently less similar to the first one than we originally thought. Therefore, if an empirical finding does not replicate, we are more concerned about our understanding of the cases than about the validity of our theory.

Whereas statistical generalization provides us with a formal (and thus somehow more objective) apparatus to evaluate the universal validity of our theories, the bottom-up strategy forces us to think about the class of intended applications on theoretical grounds. This means that we have to ask: what are the boundary conditions of our theory? In the above classroom example, following a bottom-up strategy, we would build on our preliminary understanding of the cases in one context (e.g., a public school) to search for similar and contrasting cases in other contexts (e.g., a private school). We would then re-evaluate our theoretical description of the data and explore what makes cases similar or dissimilar with regard to our theory. This enables us to expand the class of intended applications alongside with the theory.

Of course, none of these strategies is superior *per se*. Nevertheless, they rely on different assumptions and may thus be more or less adequate in different contexts. The statistical strategy relies on the assumption of a universal population and invariant measurements. This means, we assume that (a) all samples are drawn from the same population and (b) all variables refer to the same behavioral classes. If these assumptions are true, statistical generalization is valid and therefore provides a valuable tool for the testing of empirical theories. The bottom-up strategy of generalization relies on the idea that contexts may be classified as being more or less similar based on characteristics that are not part of the model being evaluated. If such a similarity relation across contexts is feasible, the bottom-up strategy is valid, as well. Depending on the strategy of generalization, replication of empirical research serves two very different purposes. Following the (top-down) principle of generalization by deduction from scientific laws, replications are empirical tests of the theory itself, and failed replications question the theory on a fundamental level. Following the (bottom-up) principle of generalization by induction to similar contexts, replications are a means to explore the boundary conditions of a theory. Consequently, failed replications question the scope of the theory and help to shape the set of intended applications.

## Conclusion

We have argued that quantitative and qualitative research are best understood by means of the structure of the employed models. Quantitative science mainly relies on variable-based models and usually employs a top-down strategy of generalization from an abstract population to individual cases. Qualitative science prefers case-based models and usually employs a bottom-up strategy of generalization. We further showed that failed replications have very different implications depending on the underlying strategy of generalization. Whereas in the top-down strategy, replications are used to test the universal validity of a model, in the bottom-up strategy, replications are used to explore the scope of a model. We will now address the implications of this analysis for psychological research with regard to the problem of replicability.

Modern day psychology almost exclusively follows a top-down strategy of generalization. Given the quantitative background of most psychological theories, this is hardly surprising. Following the general structure of variable-based models, the individual case is not the focus of the analysis. Instead, scientific laws are stated on the level of an abstract population. Therefore, when applying the theory to a new context, a statistical sampling model seems to be the natural consequence. However, this is not the only possible strategy. From a logical point of view, there is no reason to assume that a quantitative law like ∀_*i*_ : *y*_*i*_ = *f*(*x*_*i*_) implies that the law is necessarily true, i.e.,: □(∀_*i*_ : *y*_*i*_ = *f*(*x*_*i*_)). Instead, one might just as well define the scope of the theory following an inductive strategy.^8^ Formally, this would correspond to the assumption that the observed law is *possibly* true, i.e.,: ◇(∀_*i*_ : *y*_*i*_ = *f*(*x*_*i*_)). For example, we may discover a functional relation between “engagement” and “distraction” without referring to an abstract universal population of students. Instead, we may hypothesize under which conditions this functional relation may be valid and use these assumptions to inductively generalize to other cases.

If we take this seriously, this would require us to specify the intended applications of the theory: in which contexts do we expect the theory to hold? Or, equivalently, what are the boundary conditions of the theory? These boundary conditions may be specified either intensionally, i.e., by giving external criteria for contexts being similar enough to the ones already studied to expect a successful application of the theory. Or they may be specified extensionally, by enumerating the contexts where the theory has already been shown to be valid. These boundary conditions need not be restricted to the population we refer to, but include all kinds of contextual factors. Therefore, adopting a bottom-up strategy, we are forced to think about these factors and make them an integral part of our theories.

In fact, there is good reason to believe that bottom-up generalization may be more adequate in many psychological studies. Apart from the pitfalls associated with statistical generalization that have been extensively discussed in recent years (e.g., p-hacking, underpowered studies, publication bias), it is worth reflecting on whether the underlying assumptions are met in a particular context. For example, many samples used in experimental psychology are not randomly drawn from a large population, but are convenience samples. If we use statistical models with non-random samples, we have to assume that the observations vary *as if* drawn from a random sample. This may indeed be the case for randomized experiments, because all variation between the experimental conditions apart from the independent variable will be random due to the randomization procedure. In this case, a classical significance test may be regarded as an approximation to a randomization test (Edgington and Onghena, 2007). However, if we interpret a significance test as an approximate randomization test, we test not for generalization but for internal validity. Hence, even if we use statistical significance tests when assumptions about random sampling are violated, we still have to use a different strategy of generalization. This issue has been discussed in the context of small-N studies, where variable-based models are applied to very small samples, sometimes consisting of only one individual (Dugard et al., 2012). The bottom-up strategy of generalization that is employed by qualitative researchers, provides such an alternative.

Another important issue in this context is the question of measurement invariance. If we construct a variable-based model in one context, the variables refer to those behaviors that constitute the underlying empirical relational structure. For example, we may construct an abstract measure of “distraction” using the observed behaviors in a certain context. We will then use the term “distraction” as a theoretical term referring to the variable we have just constructed to represent the underlying empirical relational structure. Let us now imagine we apply this theory to a new context. Even if the individuals in our new context are part of the same population, we may still get into trouble if the observed behaviors differ from those used in the original study. How do we know whether these behaviors constitute the same variable? We have to ensure that in any new context, our measures are valid for the variables in our theory. Without a proper measurement model, this will be hard to achieve (Buntins et al., 2017). Again, we are faced with the necessity to think of the boundary conditions of our theories. In which contexts (i.e., for which sets of individuals and behaviors) do we expect our theory to work?

If we follow the rationale of inductive generalization, we can explore the boundary conditions of a theory with every new empirical study. We thus widen the scope of our theory by comparing successful applications in different contexts and unsuccessful applications in similar contexts. This may ultimately lead to a more general theory, maybe even one of universal scope. However, unless we have such a general theory, we might be better off, if we treat unsuccessful replications not as a sign of failure, but as a chance to learn.

## Author Contributions

MB conceived the original idea and wrote the first draft of the paper. MS helped to further elaborate and scrutinize the arguments. All authors contributed to the final version of the manuscript.

## Conflict of Interest

The authors declare that the research was conducted in the absence of any commercial or financial relationships that could be construed as a potential conflict of interest.

## Footnotes

^1^A person × behavior matrix constitutes a very simple relational structure that is common in psychological research. This is why it is chosen here as a minimal example. However, more complex structures are possible, e.g., by relating individuals to behaviors over time, with individuals nested within groups etc. For a systematic overview, compare Coombs (1964).

^2^This notion of empirical content applies only to deterministic models. The empirical content of a probabilistic model consists in the probability distribution over all possible empirical structures.

^3^For example, neither the SAGE Handbook of qualitative data analysis edited by Flick (2014) nor the Oxford Handbook of Qualitative Research edited by Leavy (2014) mention formal approaches to category formation.

^4^Note also that the described structure is empirically richer than a nominal scale. Therefore, a reduction of qualitative category formation to be a special (and somehow trivial) kind of measurement is not adequate.

^5^It is possible to extend this notion of empirical content to the probabilistic case (this would correspond to applying a latent class analysis). But, since qualitative research usually does not rely on formal algorithms (neither deterministic nor probabilistic), there is currently little practical use of such a concept.

^6^We do not elaborate on abductive reasoning here, since, given an empirical relational structure, the concept can be applied to both types of models in the same way (Schurz, 2008). One could argue that the underlying relational structure is not given a priori but has to be constructed by the researcher and will itself be influenced by theoretical expectations. Therefore, abductive reasoning may be necessary to establish an empirical relational structure in the first place.

^7^We shall not elaborate on the metaphysical meaning of possible worlds here, since we are only concerned with empirical theories [but see Tooley (1999), for an overview].

^8^Of course, this also means that it would be equally reasonable to employ a top-down strategy of generalization using a case-based model by postulating that □(∃_*i*_ : *XYZ*_*i*_). The implications for case-based models are certainly worth exploring, but lie beyond the scope of this article.
